# Comparison of two blunt dissection sheath insertion techniques in peripherally inserted central catheter placement: a randomized non-inferiority trial

**DOI:** 10.3389/fmed.2026.1921555

**Published:** 2026-09-24

**Authors:** Dongting Xu, Sibei Wan, Zhongling Yang, Yifan Jiang, Congcong Cao, Mengru Liu, Yan Wang, Yanping Zhuang, Hong Xing, Jingjue Mao, Ying Gu, Fang Fang

**Affiliations:** 1 Department of Nursing, Shanghai General Hospital Affiliated to Shanghai Jiao Tong University School of Medicine, Shanghai, China; 2 School of Medicine, Tongji University, Shanghai, China; 3 Department of Nursing, Shanghai Chongming District Infectious Disease Hospital, Shanghai, China; 4 School of Nursing, Bengbu Medical University, Bengbu, China; 5 Department of Nursing, Tongji Hospital Affiliated to Tongji University, Shanghai, China

**Keywords:** complications, modified Seldinger technique, peripherally inserted central catheter, PICC, sheath insertion

## Abstract

**Objective:**

To determine whether the one-step sheath insertion technique is non-inferior to the conventional two-step technique with respect to first-attempt sheath insertion success in adults undergoing peripherally inserted central catheter (PICC) placement.

**Methods:**

This prospective, single-center, outcome-assessor-blinded, randomized non-inferiority trial included 500 adult patients undergoing PICC placement, who were randomly assigned 1:1 to the one-step or two-step sheath insertion group. The primary outcome was first-attempt sheath insertion success, with a prespecified non-inferiority margin of −5 percentage points. Secondary outcomes included intraoperative and 24-hour puncture-site bleeding, insertion-site infection, and phlebitis within 7 days.

**Results:**

First-attempt sheath insertion was successful in 92.0% (230/250) of patients in the one-step group and 95.6% (239/250) in the two-step group. The absolute risk difference was −3.6 percentage points (95% CI, −7.8 to 0.6), and the lower confidence bound crossed the prespecified non-inferiority margin; therefore, non-inferiority was not demonstrated. Intraoperative puncture-site bleeding was less severe in the one-step group (*P* < 0.001), although this was a secondary outcome based on semi-quantitative assessment. No significant between-group difference was observed in bleeding at 24 h (*P* = 0.623). Insertion-site infection occurred in 0.4% of patients in each group, and phlebitis occurred in 1.2% and 0.8% of patients in the one-step and two-step groups, respectively.

**Conclusion:**

The one-step sheath insertion technique did not demonstrate non-inferiority to the conventional two-step technique for first-attempt sheath insertion success. Although less severe intraoperative puncture-site bleeding was observed with the one-step technique, this secondary finding should be interpreted cautiously. The present findings do not support routine replacement of the two-step technique.

## 1 Introduction

Peripherally inserted central catheters (PICCs) are vascular access devices inserted through a peripheral vein, with the catheter tip positioned in the central venous system, according to the standardized nomenclature proposed by the NAVIGATE project (1). Current infusion therapy standards emphasize standardized vascular assessment, ultrasound-guided puncture, aseptic technique, operator competency, and catheter maintenance to reduce PICC-related complications (2). Among current insertion approaches, the ultrasound-guided modified Seldinger technique (MST) is widely used because it enables real-time vascular visualization and guidewire-assisted catheterization (3). However, variation remains in the sequence of tract dilation and introducer sheath insertion, and the clinical implications of these technical differences have not been adequately evaluated (4). Existing recommendations provide limited specific guidance regarding the optimal sequence of dilator and peel-away sheath insertion during PICC placement.

In the conventional two-step sheath insertion technique, the dilator is first advanced over the guidewire to pre-dilate the puncture tract, withdrawn, and subsequently reassembled with the peel-away sheath for reinsertion (5). In contrast, the one-step sheath insertion technique maintains the dilator and sheath as an assembled unit, allowing tract dilation followed directly by sheath advancement. By eliminating withdrawal, reassembly, flushing, and repeated advancement, the one-step sheath insertion technique involves fewer procedural manipulations, providing a clinical rationale for investigating whether it is associated with less puncture-site bleeding. However, because separate pre-dilation is omitted, it remains uncertain whether the one-step sheath insertion technique can maintain an acceptable first-attempt sheath insertion success rate. Therefore, first-attempt sheath insertion success was selected as the primary measure of technical efficacy, while puncture-site bleeding was evaluated as a secondary outcome reflecting the potential effect of reduced procedural manipulation.

In clinical practice, variations in sheath insertion sequence may reflect local protocols or operator preference rather than evidence-based recommendations. Evidence directly comparing these sheath insertion sequences remains limited. A previous case series evaluating an optimized two-stage sheath-dilator insertion technique included only four patients and could not provide robust comparative evidence (6). Another study compared conventional and modified Seldinger techniques in neonatal PICC placement but did not specifically evaluate sheath insertion sequence in adults (5). Although variations in sheath insertion practice exist clinically, the one-step sheath insertion technique should be regarded as a procedural variation rather than an established standard approach to PICC placement. Thus, whether the procedural simplification offered by the one-step sheath insertion technique can be achieved without a clinically unacceptable reduction in first-attempt sheath insertion success rate remains uncertain.

A non-inferiority design was considered appropriate because the anticipated clinical benefit of the one-step sheath insertion technique lies primarily in reducing procedural manipulation and puncture-site bleeding rather than improving technical success itself. Accordingly, a prospective, randomized, outcome-assessor-blinded, non-inferiority trial was conducted to compare the one-step and conventional two-step sheath insertion techniques in adults undergoing PICC placement. The primary objective was to determine whether the one-step sheath insertion technique was non-inferior to the two-step sheath insertion technique in first-attempt sheath insertion success rate. Secondary outcomes included intraoperative and 24-hour puncture-site bleeding and the incidence of insertion-site infection and phlebitis within 7 days. It was hypothesized that the one-step sheath insertion technique would be non-inferior to the two-step sheath insertion technique in first-attempt sheath insertion success rate, while being associated with less puncture-site bleeding.

## 2 Methods

### 2.1 Study design and participants

This study was a prospective, single-center, parallel-group, 1:1 allocation, outcome-assessor-blinded, non-inferiority randomized controlled trial. A single-center design was adopted to ensure consistency in operator training, catheterization procedures, equipment, and outcome assessment during this initial prospective evaluation of the two sheath insertion techniques. The study was conducted at Shanghai General Hospital, a tertiary grade A hospital. The study protocol (Version 1.0) was approved by the Ethics Committee of Shanghai General Hospital (approval number: [2025]158). Following ethics approval, the trial was registered with the Chinese Clinical Trial Registry on November 24, 2025 (ChiCTR2500113045). Formal participant enrollment commenced after trial registration in November 2025 and continued through June 2026. All participants provided written informed consent before enrollment. The reporting follows the CONSORT 2010 statement and its extension for non-inferiority randomized trials.

Adult inpatients scheduled for PICC placement were screened for eligibility and enrolled after meeting the predefined eligibility criteria and providing written informed consent. PICC placement was clinically indicated for patients requiring medium- to long-term intravenous therapy, including chemotherapy, parenteral nutrition, prolonged intravenous medication, or other clinically indicated long-term venous access. The inclusion criteria were: (1) normal coagulation function; (2) age ≥18 years; (3) ability to communicate verbally and cooperate with catheter placement and follow-up assessments; (4) voluntary participation and signed informed consent. The exclusion criteria were: (1) unconsciousness or inability to cooperate with the procedure and follow-up; (2) skin breakdown, local infection, scarring, or other conditions at the intended puncture site that could interfere with catheterization; (3) thrombosis, vascular obstruction, or other anatomical abnormalities involving the intended catheterization vein; (4) severe coagulation abnormalities or thrombocytopenia that increased the risk of bleeding; (5) known contraindications to PICC placement; (6) a clinical requirement for a tunneled catheter, including a tunneled PICC or an extended subcutaneous route; and (7) any other conditions considered unsuitable for study participation by the investigator.

### 2.2 Materials, operators, and periprocedural management

All participants in both groups received the same 4-Fr single-lumen PowerPICC SOLO catheter and a 7-cm, 4.5-Fr peel-away introducer sheath (Bard Medical Inc., USA). A Site Rite 8 vascular ultrasound system and a disposable central venous catheter insertion kit were used in both groups. All devices were manufactured by Bard Medical Inc. (USA). Thus, catheter lumen number, catheter diameter, and introducer sheath size were identical between the two groups.

All catheterization procedures were performed by three intravenous therapy specialist nurses from the study hospital who held PICC placement certification and performed a minimum of 500 PICC insertions annually. Prior to the initiation of the study, all operators received standardized training covering the inclusion and exclusion criteria, the standardized workflow for the two sheath insertion techniques, criteria for blood loss assessment, complication monitoring, and instructions for completing the case report forms.

Before catheterization, patients were routinely assessed for coagulation function, platelet count, the condition of the limb on the puncture side, and the indications for PICC placement. Ultrasound was used to assess vessel diameter, depth, trajectory, and surrounding anatomy. The basilic vein was preferred as the primary target based on its larger diameter and straight course; when unsuitable, the brachial or cephalic vein was considered as an alternative, guided by real-time ultrasound assessment to ensure adequate vessel diameter and depth. In both groups, skin antisepsis, draping, ultrasound-guided venipuncture, guidewire insertion, local anesthesia, catheter insertion, fixation, and dressing coverage were performed in accordance with aseptic protocols. All ultrasound-guided punctures were performed using a standardized protocol. Catheter tip position was confirmed by chest radiography, with the target location being the junction of the lower one-third of the superior vena cava and the right atrium.

### 2.3 Randomization and blinding

A computer-generated random allocation sequence with a permuted block design using variable block sizes of 6 was used to assign participants in a 1:1 ratio to the two-step sheath insertion group and the one-step sheath insertion group; no stratification was applied. The random sequence was generated by a researcher who was not involved in clinical procedures, outcome assessment, or statistical analysis, using SPSS 22.0, and this researcher was blinded to participant characteristics. Allocation concealment was achieved with sequentially numbered, sealed, opaque envelopes. After patients provided informed consent and completed baseline assessments, the designated catheterization nurse opened the envelope in numerical order before the procedure and performed the assigned intervention.

Because the two sheath insertion techniques differ in procedural steps, the catheterization nurses performing the procedures could not be blinded to group assignment. The dilators and peel-away sheaths used in both groups were identical in appearance, making it impossible for patients to distinguish between the two groups. Outcome measures, including the first-attempt sheath insertion success rate, intraoperative and postoperative puncture-site bleeding, insertion-site infection, and phlebitis, were assessed by trained research nurses who were not involved in randomization or catheterization procedures. The statistician who performed the data analysis was also blinded to group assignment. These measures minimized detection and analysis bias.

### 2.4 Interventions

The devices used for the two sheath insertion techniques are shown in Figure 1A, including the assembled introducer, the separated dilator and peel-away sheath, the puncture needle, and the guidewire.

**Figure 1:**
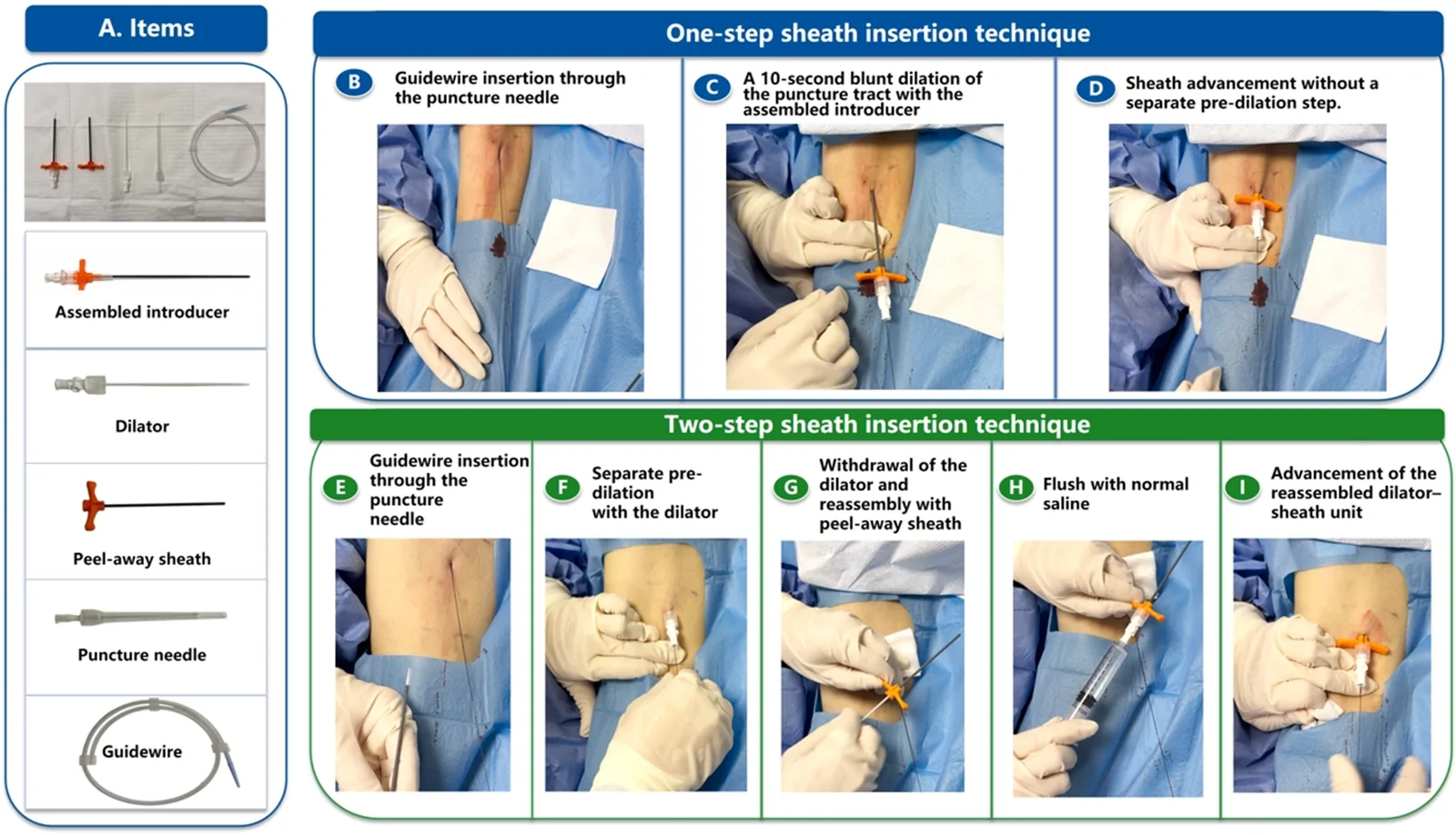
Components and procedural sequences of the one-step and two-step sheath insertion techniques. **(A)** Devices used for sheath insertion. **(B–D)** One-step technique: guidewire insertion, 10-second blunt dilation with the assembled introducer, and sheath advancement without separate pre-dilation. **(E–I)** Two-step technique: guidewire insertion, separate pre-dilation, dilator withdrawal and reassembly with the peel-away sheath, saline flushing, and advancement of the reassembled dilator–sheath unit.

The two-step sheath insertion group received the two-step sheath insertion technique. After routine skin antisepsis and draping, ultrasound-guided venipuncture was performed, followed by guidewire insertion through the puncture needle (Figure 1E). Following local anesthesia with 2% lidocaine, the peel-away sheath and dilator were separated. The dilator was first advanced over the guidewire to pre-dilate the puncture tract and the venous entry site (Figure 1F). The dilator was then withdrawn and reassembled with the peel-away sheath (Figure 1G), after which the reassembled dilator–sheath unit was flushed with normal saline (Figure 1H). The operator then stretched the skin below the puncture site with the left hand and advanced the reassembled dilator–sheath unit into the vein over the guidewire using the standardized “hammer-drill” technique (Figure 1I).

The one-step sheath insertion group received the one-step sheath insertion technique. After routine skin antisepsis and draping, ultrasound-guided venipuncture was performed, followed by guidewire insertion through the puncture needle (Figure 1B). Following local anesthesia with 2% lidocaine, the dilator and peel-away sheath were not separated. The tapered tip of the dilator, which protrudes from the distal end of the peel-away sheath, was introduced into the puncture site over the guidewire and held in place for 10 s to achieve blunt dilation of the puncture tract and subcutaneous tissue in Figure 1C. Subsequently, without a separate pre-dilation step or withdrawal and reassembly of the dilator, the operator stretched the skin below the puncture site with the left hand and advanced the assembled dilator–sheath unit directly into the vein over the guidewire using the standardized “hammer-drill” technique (Figure 1D).

The one-step and two-step sheath insertion techniques are demonstrated in Supplementary Videos 1 and 2, respectively. In both groups, the introducer was advanced through the original venipuncture site. Neither an extended subcutaneous route nor a pseudotunneling technique was used, and no additional skin exit site or subcutaneous tunnel was created. Therefore, the blunt dilation of the puncture tract and adjacent subcutaneous tissue described above did not constitute pseudotunneling, and pseudotunnel length was not applicable.

In both groups, sheath advancement was performed using the standardized “hammer-drill” technique, which consisted of alternating push-and-pause movements with consistent force application across all operators. The 10-second blunt dilation period in the one-step group was timed using a stopwatch to ensure standardization. Protocol deviations were defined *a priori* as: (1) switching from the assigned sheath insertion method to the alternative method during the procedure, (2) performing an additional skin incision beyond the initial puncture site, or (3) repeating venipuncture after guidewire failure. Crossover criteria were prespecified: if the assigned sheath could not be advanced after two attempts, the operator switched to the alternative technique as the rescue strategy. No additional skin incisions or repeat venipunctures were required in either group.

In both groups, after the peel-away sheath was successfully advanced into the vein, the guidewire and dilator were withdrawn, and the catheter was slowly advanced through the peel-away sheath. Once the catheter was fully inserted into the body, the peel-away sheath was withdrawn and peeled away. The catheter was then adjusted to the 0 cm mark, a needleless injection cap was connected, and the catheter was flushed and locked. The catheter was secured using a StatLock fixation device, and the puncture site was covered with a 3 cm × 5 cm, 24-ply sterile gauze pad, over which a sterile transparent dressing was applied. After catheter placement, patients in both groups were instructed to avoid strenuous activity of the upper limb on the catheterized side within 24 h. All patients in both groups received the same treatment and nursing care following catheter placement.

### 2.5 Outcome measures

The primary outcome measure was the first-attempt sheath insertion success rate. First-attempt sheath insertion success was defined as the successful insertion of the peel-away sheath into the vein in a single attempt after local anesthesia, in accordance with the randomly assigned sheath insertion technique, without changing the assigned technique, without additional skin incision, and without re-puncture. Cases requiring a change to an alternative technique, repeated sheath insertion, re-puncture, or additional skin incision were classified as first-attempt sheath insertion failure.

Secondary outcome measures included intraoperative puncture-site bleeding, puncture-site bleeding within 24 h after catheter placement, insertion-site infection within 7 days after catheter placement, and clinically assessed PICC-associated phlebitis within 7 days after catheter placement.

Intraoperative puncture-site bleeding was defined as the amount of bleeding from the puncture site occurring between the completion of local anesthesia with 2% lidocaine and the insertion of the peel-away sheath into the vein. After the completion of local anesthesia, a fresh 5 cm × 5 cm, 4-ply sterile gauze pad was placed over the puncture site. An independent assessment nurse graded the blood loss based on the area of gauze soaked: a soaked area less than half of one gauze pad was classified as mild bleeding; a soaked area equal to or greater than half but less than one full gauze pad was classified as moderate bleeding; and a soaked area equal to or exceeding one full gauze pad was classified as severe bleeding (7).

The gauze-based bleeding assessment used in this study was adapted from previously published methods for evaluating puncture-site bleeding after vascular access procedures. The grading criteria were defined before study initiation and applied consistently by trained research nurses who were blinded to group assignment.

Puncture-site bleeding at 24 h postoperatively was defined as the amount of bleeding observed on the gauze covering the puncture site 24 h after the completion of catheter placement, with the grading criteria identical to those for intraoperative puncture-site bleeding. Insertion-site infection was defined according to the Centers for Disease Control and Prevention (CDC) criteria as the presence of redness, swelling, heat, pain, or purulent discharge at the insertion site, or a positive bacterial culture from the insertion site. This definition refers to local insertion-site infection, not catheter-related bloodstream infection (CRBSI), which was not assessed in this study due to the short 7-day follow-up period. Phlebitis was clinically assessed within 7 days after PICC placement according to the prespecified study criteria and was defined as the presence of a palpable cord-like reddened area, induration, or pain along the venous trajectory, with a phlebitis score of ≥2. Etiological subtype data were not collected; therefore, phlebitis was not further classified as mechanical, chemical, or infectious. The 7-day follow-up period was selected to capture early local complications. Outcomes requiring imaging-based or longer-term surveillance, including catheter-related thrombosis and CRBSI, were beyond the scope of this study (2). Within 7 days after catheter placement, a trained research nurse who was not involved in group assignment or the procedure assessed and recorded findings for the puncture site and the catheterized limb daily at 08:00.

### 2.6 Sample size estimation

The sample size was calculated for a 1:1 parallel-group non-inferiority randomized controlled trial using the first-attempt sheath insertion success rate as the primary outcome. The parameters were established based on data from a pilot study conducted at the study center from January to February 2023. The pilot study enrolled 60 adult patients meeting the inclusion and exclusion criteria for PICC placement, with 30 patients in the two-step sheath insertion group and 30 patients in the one-step sheath insertion group. In the two-step sheath insertion group, first-attempt sheath insertion was successful in 29 cases, yielding a success rate of 96.7%; in the one-step sheath insertion group, first-attempt sheath insertion was successful in 28 cases, yielding a success rate of 93.3%The non-inferiority margin was set at −5 percentage points based on a dual approach. First, a review of historical literature on sheath insertion techniques in PICC placement indicated that the two-step technique achieves first-attempt success rates of approximately 95%–97%, with the absolute risk reduction relative to non-dilated puncture estimated at 10%–12%. According to regulatory guidance (FDA, 2016), preserving at least 50% of the active control effect is considered an acceptable criterion for non-inferiority (8). Based on half of the historical 10% risk reduction, a non-inferiority margin of −5 percentage points was derived. Second, a consensus was reached among five senior vascular access nurses (each performing >500 PICC insertions annually) that a 5% absolute difference in success rate is clinically acceptable and would not outweigh the bleeding-related benefits of the one-step technique. Sample size calculation was performed using PASS 15.0 (NCSS, LLC, Kaysville, UT, USA) for a binary non-inferiority test. The following assumptions were used: two-sided significance level *α* = 0.05, power 1−*β* = 0.80, control group success rate of 96.7% derived from the pilot study (30 patients in the two-step group, with 29 successful first-attempt sheath insertions), non-inferiority margin of −5 percentage points, and equal allocation ratio (1:1). The unadjusted sample size was 224 patients per group. Accounting for a 10% attrition rate during the 7-day follow-up period, the target sample size was 249 patients per group, totaling 498 patients. A total of 500 patients were ultimately enrolled to allow for potential exclusions. The primary non-inferiority analysis was performed using the intention-to-treat (ITT) population. A sensitivity analysis using the per-protocol population was planned.

### 2.7 Statistical analysis

Statistical analyses were performed using SPSS 22.0 (IBM Corp., Armonk, NY, USA). Continuous variables were tested for normality using the Shapiro–Wilk test. Variables following a normal distribution were presented as mean ± standard deviation and compared between groups using the independent samples t-test; those not normally distributed were presented as median and interquartile range and compared using the Mann–Whitney U test. Categorical variables were presented as frequencies and percentages and compared using the chi-square test or Fisher's exact test, as appropriate. Intraoperative and 24-hour postoperative puncture site bleeding were treated as ordinal categorical variables and compared between groups using the Mann–Whitney U test.

Primary non-inferiority analysis. The primary analysis was performed on the intention-to-treat (ITT) population, which included all randomized participants according to their original group assignment, regardless of the actual procedure received. The difference in the first-attempt sheath insertion success rate between the one-step and two-step groups, and its two-sided 95% confidence interval (CI), were calculated. The 95% CI for the risk difference (one-step minus two-step) was computed using the Wilson score method without continuity correction (Newcombe–Wilson method), which is recommended for binary outcomes and provides better coverage properties than the Wald method, particularly when success rates are high (>90%) (9). Non-inferiority was declared if the lower bound of the 95% CI was greater than the pre-specified non-inferiority margin of −5 percentage points.

#### 2.7.1 Multiplicity

Since this trial had only one primary endpoint, no adjustment for multiple comparisons (e.g., Bonferroni correction) was applied. Secondary outcomes were considered exploratory, and their *P* values are reported without multiplicity adjustment.

#### 2.7.2 Protocol deviations

Protocol deviations were defined *a priori* as described in Section 2.4. According to the prespecified analysis plan, participants with protocol deviations were intended to be retained in the ITT analysis and counted as first-attempt sheath insertion failures.

#### 2.7.3 Missing data

The primary outcome (first-attempt sheath insertion success) was assessed at the time of the procedure, with complete data available for all 500 participants (0% missing). For secondary outcomes at 7 days post-catheterization, the missing data rate was less than 1%, which was below the pre-specified 5% threshold; therefore, complete-case analysis was used without multiple imputation.

#### 2.7.4 Sensitivity analyses

A per-protocol sensitivity analysis was considered; however, it could not be performed because protocol deviations were not systematically captured. Therefore, the non-inferiority conclusion was based on the ITT analysis alone, and the absence of a formal PP sensitivity analysis should be considered when interpreting the robustness of the findings. This limitation has been acknowledged in the Limitations section.

#### 2.7.5 CONSORT compliance

This study was conducted and reported in accordance with the CONSORT 2010 Statement and its extension for non-inferiority randomized trials. A completed CONSORT checklist is provided as Supplementary Table S1. All statistical tests, except for the primary non-inferiority analysis, were two-sided, and a *P* value ≤ 0.05 was considered statistically significant.

## 3 Results

A total of 500 patients were enrolled and randomized 1:1 to the one-step group (*n* = 250) or the two-step group (*n* = 250). No patients were excluded after randomization, and all 500 participants completed the 7-day follow-up (500/500, 100%). No treatment crossovers were documented (0/500, 0%), and no participants were lost to follow-up (0/500, 0%). However, minor deviations from the procedural protocol were not prospectively recorded in a dedicated field of the case report form. Therefore, the overall protocol-deviation rate could not be reliably calculated, and a formal per-protocol population could not be defined. Consequently, the primary non-inferiority analysis was based on the ITT population. The CONSORT flow diagram is presented in Figure 2.

**Figure 2:**
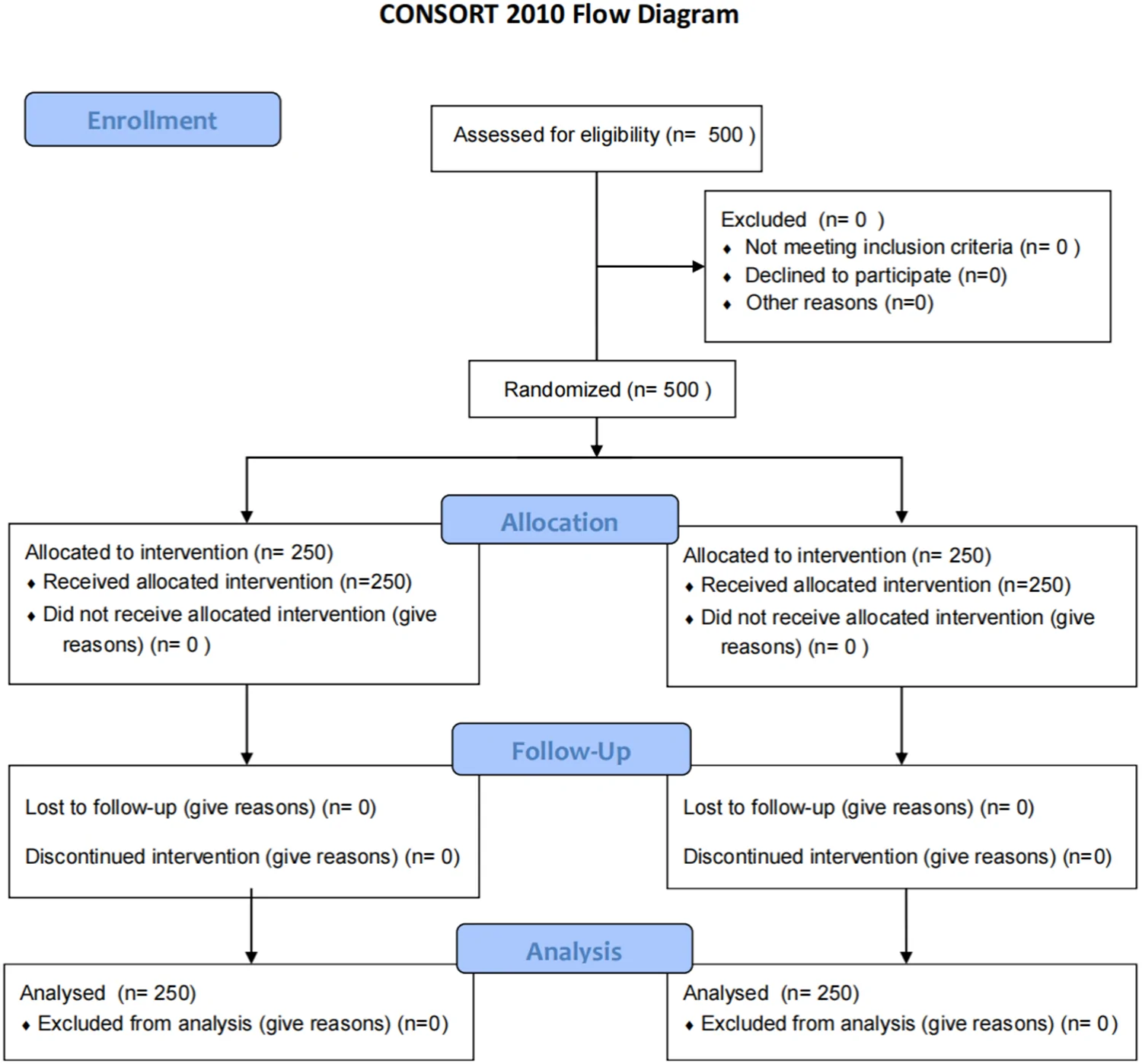
CONSORT flow diagram.

### 3.1 Comparison of baseline data between the two groups

Baseline demographic and clinical characteristics of the two groups are presented in Table 1. The analysis population for baseline characteristics was the ITT population (all 500 randomized participants). The two groups were well balanced with respect to age, sex, BMI, coagulation parameters (PLT, PT, INR), primary diagnosis, catheterization side, and physical activity status. Formal statistical comparisons showed no significant intergroup differences (all *P* > 0.05), and the two groups were clinically comparable: mean age differed by less than 2 years (56.7 vs. 58.5 years), BMI distributions were nearly identical (median 22.80 vs. 22.86), and the proportions of major diagnostic categories were similar across groups. This clinical balance supports the validity of the subsequent comparative analyses.

**Table 1:** Baseline demographic and clinical characteristics of participants in the one-step and two-step sheath insertion groups.

Variable	One-step sheath insertion group (*n* = 250)	Two-step sheath insertion group (*n* = 250)	Statistic	*P*
Height (cm)	166.83 ± 7.92	166.77 ± 7.77	0.077	0.939
Weight (kg)	64.55 ± 12.47	63.79 ± 11.76	0.694	0.488
BMI	22.80(20.45，25.35)	22.86(20.05，25.24)	0.670	0.503
Age (years)	56.67 ± 14.88	58.51 ± 14.29	−1.410	0.159
PLT within 1 week before PICC insertion	184.42 ± 31.79	186.35 ± 29.69	−0.701	0.484
PT within 1 week before PICC insertion	12.33(11.62，13.18)	12.49(11.69，13.16)	−0.796	0.426
INR within 1 week before PICC insertion	1.03 ± 0.09	1.01 ± 0.08	0.765	0.445
Sex [n (%)]			0.716	0.397
Male	159(63.6)	168(67.2)		
Female	91(36.4)	82(32.8)		
Primary diagnosis [n (%)]			4.248	0.514
Head and neck tumors	21(8.4)	33(13.2)		
Gastrointestinal tumors	71(28.4)	77(30.8)		
Respiratory tumors	49(19.6)	43(17.2)		
Genitourinary tumors	29(11.6)	23(9.2)		
Hematologic malignancies	73(29.2)	68(27.2)		
Non-malignant	7(2.8)	6(2.4)		
Regular physical activity [n (%)]			0.229	0.632
No	227(90.8)	230(92.0)		
Yes	23(9.2)	20(8.0)		
Insertion site [n (%)]			2.801	0.094
Right arm	242(96.8)	234(93.6)		
Left arm	8(3.2)	16(6.4)		
Manual labor [n (%)]			0.343	0.558
No	226(90.4)	222(88.8)		
Yes	24(9.6)	28(11.2)		

### 3.2 Comparison of PICC placement-related outcomes between the two groups

Reasons for first-attempt sheath insertion failure were documented. In the one-step group, 20 failures occurred: 14 due to sheath advancement resistance, 4 due to guidewire deviation, and 2 due to vessel spasm. In the two-step group, 11 failures occurred: 6 due to sheath advancement resistance, 3 due to guidewire deviation, and 2 due to vessel spasm.

Primary outcome: first-attempt sheath insertion success. The first-attempt sheath insertion success rate was 92.0% (230/250) in the one-step group and 95.6% (239/250) in the two-step group. The absolute risk difference (one-step minus two-step) was −3.6 percentage points (95% CI: −7.8 to 0.6 percentage points). The lower bound of the 95% CI fell below the pre-specified non-inferiority margin of −5 percentage points. Therefore, non-inferiority of the one-step technique compared with the two-step technique was not demonstrated. A forest plot visualizing the non-inferiority analysis is provided in Figure 3.

**Figure 3:**
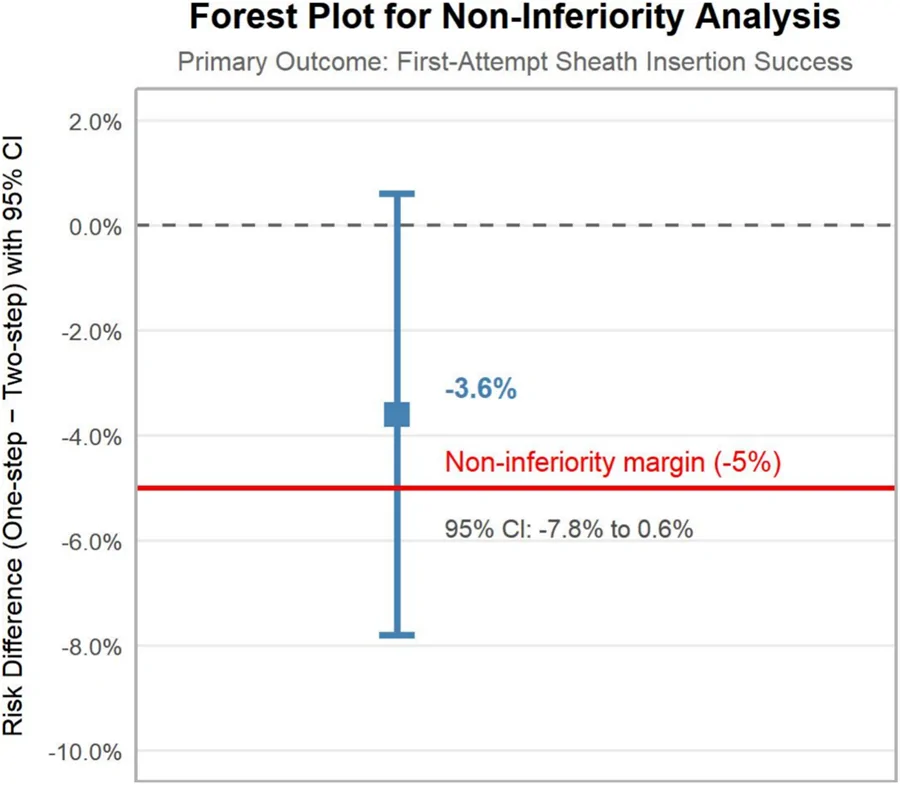
Forest plot for the non-inferiority analysis of first-attempt sheath insertion success. The risk difference was calculated as one-step minus two-step. The solid line indicates the prespecified non-inferiority margin of −5%, and the dashed line indicates no difference (RD = 0).

Comparisons of PICC catheterization-related outcomes between the two groups are shown in Table 2. intraoperative puncture-site bleeding was significantly lower in the one-step group than in the two-step group (*P* < 0.001), with the majority of one-step patients (99.2%) having mild bleeding, whereas the two-step group had a higher proportion of moderate and severe bleeding (36.4% and 18.8%, respectively). Detailed bleeding severity distributions are presented in Table 2.

**Table 2:** Comparison of PICC insertion-related outcomes between the one-step and two-step sheath insertion groups.

Variable	One-step sheath insertion group (*n* = 250)	Two-step sheath insertion group (*n* = 250)	Statistic	*P*
PICC-related complications: insertion-site infection and phlebitis within 7 days [n (%)]			<0.001	1.000*
No	246(98.4)	247(98.8)		
Yes	4(1.6)	3(1.2)		
First-attempt sheath insertion success rate [n (%)]			2.786	0.095
Failure	20(8.0)	11(4.4)		
Success	230(92.0)	239(95.6)		
Intraoperative puncture-site bleeding [n (%)]				<0.001**
Mild	248(99.2)	112(44.8)		
Moderate	0(0.0)	91(36.4)		
Severe	2(0.8)	47(18.8)		
Puncture-site bleeding within 24 h after PICC insertion [n (%)]				0.623*
Mild	249(99.6)	247(98.8)		
Moderate	1(0.4)	3(1.2)		
Severe	0(0.0)	0(0.0)		

* Fisher's exact test for categorical variables.

** Mann–Whitney U test for ordinal bleeding severity data.

At 24 h after PICC placement, there was no statistically significant intergroup difference in puncture-site bleeding severity (P* = 0.623). Detailed bleeding severity distributions are presented in Table 2.

Within 7 days after catheterization, the combined incidence of insertion-site infection and/or clinically assessed phlebitis was 1.6% (4/250) in the one-step group and 1.2% (3/250) in the two-step group, with no statistically significant between-group difference (*P* = 1.000; Table 2). When the two complications were considered separately for descriptive purposes, insertion-site infection occurred in 1 patient (0.4%) in each group, whereas clinically assessed phlebitis occurred in 3 patients (1.2%) in the one-step group and 2 patients (0.8%) in the two-step group. Because of the small number of individual events, no separate inferential comparisons or effect estimates were reported for insertion-site infection or phlebitis.

For secondary outcomes, the between-group difference in intraoperative puncture-site bleeding was statistically significant (P* < 0.001), favoring the one-step group. Due to the ordinal nature of the bleeding outcome, effect estimates with confidence intervals were not calculated.

## 4 Discussion

### 4.1 First-attempt sheath insertion success

The primary analysis did not demonstrate non-inferiority of the one-step sheath insertion technique for first-attempt sheath insertion success. Because the lower confidence bound crossed the prespecified non-inferiority margin, a clinically relevant reduction in technical success could not be excluded. However, failure to demonstrate non-inferiority should not be interpreted as definitive evidence that the one-step technique is inferior.

Direct evidence comparing different sheath-dilator insertion sequences remains limited. Previous PICC studies have mainly evaluated catheter tip positioning and overall catheter placement outcomes (10, 11), whereas procedural modifications have been investigated in different patient populations (5, 6).

A possible mechanical explanation is that the one-step technique omits separate tract pre-dilation before sheath advancement. Advancing the dilator and sheath as an assembled unit may encounter greater tissue resistance, potentially reducing first-attempt success in some patients. Conversely, eliminating withdrawal, reassembly, and repeated advancement may reduce local manipulation. Because insertion force, tissue resistance, and tissue trauma were not objectively measured, these explanations remain hypotheses. Operator experience may also have influenced the findings because all procedures were performed by experienced vascular access nurses at a single center (2, 12).

### 4.2 Puncture-site bleeding and short-term complications

intraoperative puncture-site bleeding was less severe with the one-step technique. One possible explanation is that reducing procedural manipulation may have contributed to less local tissue trauma. However, bleeding was assessed semi-quantitatively, and this secondary outcome should not be interpreted as evidence of reduced total blood loss or clinical superiority.

Previous studies evaluated puncture-site bleeding in relation to different PICC insertion techniques and patient populations (7, 13, 14), but they do not provide direct mechanistic evidence for the difference observed in the present study. The absence of a between-group difference at 24 h further suggests that the intraprocedural finding did not represent a sustained hemostatic benefit.

Phlebitis and insertion-site infection were uncommon during the 7-day follow-up. Because the number of events was low and phlebitis etiology was not further classified, firm conclusions regarding comparative short-term safety cannot be drawn (15, 16).

In addition to procedural modifications, adjunctive strategies aimed at improving catheter securement and exit-site protection have also been explored to reduce early bleeding-related complications. The updated SIP protocol incorporates cyanoacrylate glue as one of the strategies for minimizing complications associated with PICC insertion (16). Recent randomized clinical trials have further suggested that cyanoacrylate glue may reduce peri-catheter bleeding and early dressing-related interventions in patients undergoing midline catheter or PICC placement (17, 18). However, cyanoacrylate glue was not used in the present trial; therefore, the bleeding differences observed in this study should be interpreted as related to the sheath insertion techniques evaluated rather than adjunctive glue application. Future studies combining optimized insertion techniques with adjunctive securement strategies may help determine whether these approaches provide additional clinical benefits.

### 4.3 Clinical implications

The present findings do not support routine replacement of the conventional two-step technique because non-inferiority was not demonstrated for first-attempt sheath insertion success. Although the one-step technique may reduce procedural manipulation and immediate puncture-site bleeding, this potential benefit must be weighed against the possibility of reduced technical success. The findings do not support recommendations for specific patient subgroups. Selection of the sheath insertion technique should therefore be based on standardized vascular assessment, operator competence, and established institutional procedures (2, 19).

## 5 Limitations

This study has several limitations. First, this was a single-center trial, and all PICC insertions were performed by experienced vascular access nurses. Although this setting reduced procedural variability, it may limit the generalizability of the findings to other institutions, clinical settings, and operators with different levels of experience. In addition, blinding of operators was not feasible because of the inherent procedural differences between the two sheath insertion techniques. Operator awareness of group allocation may therefore have influenced procedural manipulation and decision-making. Although no treatment crossovers or losses to follow-up were documented, minor procedural deviations were not prospectively captured in a dedicated case report form field. Therefore, the overall protocol-deviation rate could not be quantified, and a formal per-protocol analysis could not be performed.

Second, puncture-site bleeding was assessed semi-quantitatively according to the area of gauze saturation rather than by objective measurement of blood loss or tissue trauma, which may have introduced measurement subjectivity. Procedural time, patient-reported outcomes such as pain and satisfaction, and cost or resource-utilization data were not systematically collected. Therefore, no conclusions regarding procedural efficiency, patient experience, or cost-effectiveness can be drawn from the present study. Furthermore, the study was not designed or adequately powered for subgroup analyses; therefore, potential differences among patient subgroups could not be evaluated.

Finally, follow-up was limited to 7 days, and the numbers of insertion-site infection and phlebitis events were low. Phlebitis was assessed clinically, but its etiology was not further classified as mechanical, chemical, or infectious. In addition, routine ultrasonographic screening for catheter-related thrombosis was not performed; therefore, no conclusions regarding catheter-related thrombosis can be drawn from the present data. Larger, adequately powered multicenter studies with longer follow-up and objective measures of blood loss and tissue trauma are needed to further evaluate the comparative outcomes of the two sheath insertion techniques.

## Data Availability

The raw data supporting the conclusions of this article will be made available by the authors, without undue reservation.
