# Supplementary material for: Comparison of two blunt dissection sheath insertion techniques in peripherally inserted central catheter placement: a randomized non-inferiority trial
Source: Front Med (Lausanne). 2026 Sep 24;13:1921555. doi: 10.3389/fmed.2026.1921555 (PMC13646084; doi:10.3389/fmed.2026.1921555)
Supplement: Supplementary file 1 [file Supplementaryfile1.pdf]

CONSORT Statement 2006 - Checklist for Non-inferiority and Equivalence Trials

Items to include when reporting a non-inferiority or equivalence randomized trial

PAPER SECTION And topic	Item	Descriptor	Reported on Page #
TITLE & ABSTRACT	1	How participants were allocated to interventions (*e.g.*, "random allocation", "randomized", or "randomly assigned"), *specifying that the trial is a non-inferiority or equivalence trial.*	Title & Abstract
INTRODUCTION Background	2	Scientific background and explanation of rationale, *including the rationale for using a non-inferiority or equivalence design.*	Introduction
METHODS Participants	3	Eligibility criteria for participants *(detailing whether participants in the non-inferiority or equivalence trial are similar to those in any trial(s) that established efficacy of the reference treatment)* and the settings and locations where the data were collected.	Methods
Interventions	4	Precise details of the interventions intended for each group *detailing whether the reference treatment in the non-inferiority or equivalence trial is identical (or very similar) to that in any trial(s) that established efficacy,* and how and when they were actually administered.	Methods
Objectives	5	Specific objectives and hypotheses, *including the hypothesis concerning non-inferiority or equivalence.*	Methods
Outcomes	6	Clearly defined primary and secondary outcome measures *detailing whether the outcomes in the non-inferiority or equivalence trial are identical (or very similar) to those in any trial(s) that established efficacy of the reference treatment* and, when applicable, any methods used to enhance the quality of measurements (*e.g.*, multiple observations, training of assessors).	Methods
Sample size	7	How sample size was determined *detailing whether it was calculated using a non-inferiority or equivalence criterion and specifying the margin of equivalence with the rationale for its choice.* When applicable, explanation of any interim analyses and stopping rules (*and whether related to a non-inferiority or equivalence hypothesis*).	Methods
Randomization -- Sequence generation	8	Method used to generate the random allocation sequence, including details of any restrictions (*e.g.*, blocking, stratification)	Methods
Randomization -- Allocation concealment	9	Method used to implement the random allocation sequence (*e.g.*, numbered containers or central telephone), clarifying whether the sequence was concealed until interventions were assigned.	Methods
Randomization -- Implementation	10	Who generated the allocation sequence, who enrolled participants, and who assigned participants to their groups.	Methods
Blinding (masking)	11	Whether or not participants, those administering the interventions, and those assessing the outcomes were blinded to group assignment. If done, how the success of blinding was evaluated.	Methods
Statistical methods	12	Statistical methods used to compare groups for primary outcome(s), *specifying whether a one or two-sided confidence interval approach was used.* Methods for additional analyses, such as subgroup analyses and adjusted analyses.	Methods
RESULTS Participant flow	13	Flow of participants through each stage (a diagram is strongly recommended). Specifically, for each group report the numbers of participants randomly assigned, receiving intended treatment, completing the study protocol, and analyzed for the primary outcome. Describe protocol deviations from study as planned, together with reasons.	Results
Recruitment	14	Dates defining the periods of recruitment and follow-up.	Results
Baseline data	15	Baseline demographic and clinical characteristics of each group.	Results
Numbers analyzed	16	Number of participants (denominator) in each group included in each analysis and whether the analysis was *"intention-to-treat" and/or alternative analyses were conducted.* State the results in absolute numbers when feasible (*e.g.*, 10/20, not 50%).	Results

Outcomes and estimation	17	For each primary and secondary outcome, a summary of results for each group, and the estimated effect size and its precision (*e.g.*, 95% confidence interval). *For the outcome(s) for which non-inferiority or equivalence is hypothesized, a figure showing confidence intervals and margins of equivalence may be useful.*	Results
Ancillary analyses	18	Address multiplicity by reporting any other analyses performed, including subgroup analyses and adjusted analyses, indicating those pre-specified and those exploratory.	Results
Adverse events	19	All important adverse events or side effects in each intervention group.	Results
DISCUSSION Interpretation	20	Interpretation of the results, taking into account the *non-inferiority or equivalence hypothesis and any other* study hypotheses, sources of potential bias or imprecision and the dangers associated with multiplicity of analyses and outcomes.	Discussion
Generalizability	21	Generalizability (external validity) of the trial findings.	Discussion
Overall evidence	22	General interpretation of the results in the context of current evidence.	Discussion
